# Human and vector behaviors determine exposure to *Anopheles* in Namibia

**DOI:** 10.1186/s13071-022-05563-6

**Published:** 2022-11-17

**Authors:** Tabeth Mwema, Ophilia Lukubwe, Rosalia Joseph, Deodatus Maliti, Iitula Iitula, Stark Katokele, Petrina Uusiku, Dennis Walusimbi, Sheila Barasa Ogoma, Munya Tambo, Cara Smith Gueye, Yasmin A. Williams, Elodie Vajda, Allison Tatarsky, Seth J. Eiseb, Davis R. Mumbengegwi, Neil F. Lobo

**Affiliations:** 1 University of Namibia, Windhoek, Namibia; 2 Ministry of Health and Social Services (MoHSS), Windhoek, Namibia; 3 Clinton Health Access Initiative (CHAI), Boston, MA USA; 4 Malaria Elimination Initiative, Institute of Global Health Sciences, University of California San Francisco, San Francisco, CA USA; 5 University of Notre Dame, Notre Dame, IN USA

**Keywords:** Human behavior, *Anopheles* biting behavior, Long-lasting insecticidal net use, Namibia

## Abstract

**Background:**

Although the Republic of Namibia has significantly reduced malaria transmission, regular outbreaks and persistent transmission impede progress towards elimination. Towards an understanding of the protective efficacy, as well as gaps in protection, associated with long-lasting insecticidal nets (LLINs), human and *Anopheles* behaviors were evaluated in parallel in three malaria endemic regions, Kavango East, Ohangwena and Zambezi, using the Entomological Surveillance Planning Tool to answer the question: where and when are humans being exposed to bites of *Anopheles* mosquitoes?

**Methods:**

Surveillance activities were conducted during the malaria transmission season in March 2018 for eight consecutive nights. Four sentinel structures per site were selected, and human landing catches and human behavior observations were consented to for a total of 32 collection nights per site. The selected structures were representative of local constructions (with respect to building materials and size) and were at least 100 m from each other. For each house where human landing catches were undertaken, a two-person team collected mosquitoes from 1800 to 0600 hours.

**Results:**

Surveillance revealed the presence of the primary vectors *Anopheles arabiensis*, *Anopheles gambiae* sensu stricto (s.s.) and *Anopheles funestus* s.s., along with secondary vectors (*Anopheles coustani* sensu lato and *Anopheles squamosus*), with both indoor and outdoor biting behaviors based on the site. Site-specific human behaviors considerably increased human exposure to vector biting. The interaction between local human behaviors (spatial and temporal presence alongside LLIN use) and vector behaviors (spatial and temporal host seeking), and also species composition, dictated where and when exposure to infectious bites occurred, and showed that exposure was primarily indoors in Kavango East (78.6%) and outdoors in Ohangwena (66.7%) and Zambezi (81.4%). Human behavior-adjusted exposure was significantly different from raw vector biting rate.

**Conclusions:**

Increased LLIN use may significantly increase protection and reduce exposure to malaria, but may not be enough to eliminate the disease, as gaps in protection will remain both indoors (when people are awake and not using LLINs) and outdoors. Alternative interventions are required to address these exposure gaps. Focused and question-based operational entomological surveillance together with human behavioral observations may considerably improve our understanding of transmission dynamics as well as intervention efficacy and gaps in protection.

**Graphical Abstract:**

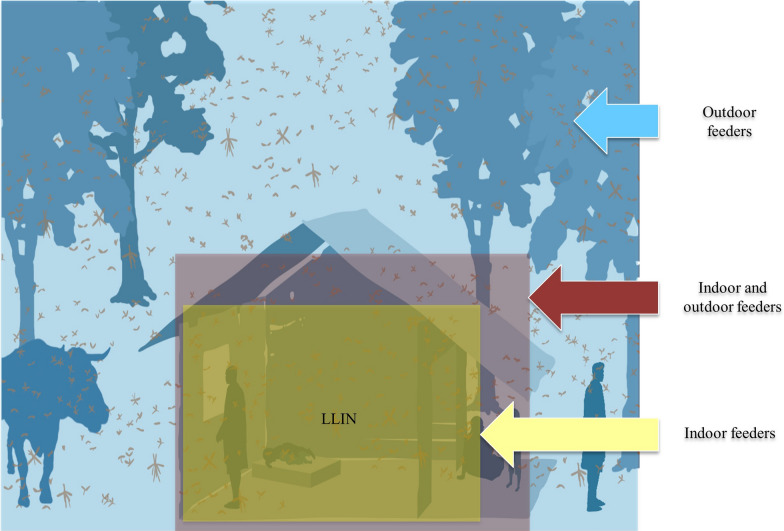

## Background

Large-scale implementation of targeted indoor residual spraying (IRS) and distribution of long-lasting insecticidal nets (LLINs), and treatment with artemisinin-based combination therapy alongside the use of rapid diagnostic tests, have resulted in Namibia being a low malaria transmission country [1, 2]. However, the target of Namibia achieving malaria elimination by 2020 was not reached due to persistent annual malaria outbreaks—especially in the northern part of the country. Baseline comprehension of the drivers of malaria transmission alongside routine surveillance is required towards achieving the optimal formulation of regionally adapted intervention strategies to address persistent low levels of transmission and prevent seasonal malaria outbreaks—important steps when zero malaria transmission is the goal [3, 4].

In Namibia, malaria transmission is seasonal, following the onset of rain, and peaks between April and May, with longer durations of higher transmission in the north-east [5]. Though disease burdens differ between regions, Kavango and Zambezi regions have the highest rates of malaria morbidity and mortality [6]. In 1990, higher than usual rainfall resulted in a severe malaria epidemic, which led to the launch of the National Vector-borne Disease Control Program (NVDCP) of the Namibian Ministry of Health and Social Services (MoHSS) [6]. In 2002, malaria was responsible for significant mortality and morbidity in Namibia, with 8.6% of hospital deaths, 26.4% of outpatient cases, as well as 21.6% of hospital admissions due to the disease [7]. The introduction of artemether-lumefantrine, improved IRS coverage (the primary vector intervention), as well as diagnosis with rapid diagnostic tests, resulted in a decline in malaria cases from an annual parasite index (API) of 62.2 per 1000 population in 2008 to 6.5 in 2011. This achievement paved the way for Namibia to become one of the eight countries in southern Africa with the potential to eliminate malaria by 2030 [2]. However, rainy season-associated malaria incidence fluctuated between an API of 1.4 in 2012 and 15.1 in 2018. In 2018, a total of 81% of malaria cases in Namibia were recorded in Kavango East, Kavango West, and Zambezi regions [6].

Since the 1960s, the main vector control intervention in Namibia has been IRS, primarily with dichlorodiphenyltrichloroethane. More recently, dichlorodiphenyltrichloroethane has been used largely on traditional structures, i.e., huts traditionally built with wood, animal dung, mud, grass and/or stones, whereas deltamethrin has been used on cement block structures [2]. Although the insecticides used in IRS do not necessarily prevent mosquitoes from biting humans, the increased daily mortality rate of mosquitoes that rest on sprayed walls reduces their population-level vectorial capacity and therefore malaria transmission. For IRS to be effective, more than 80% of the houses in an area need to be sprayed [8]. However, low IRS coverage in 2008 (48.9% coverage in at-risk areas) resulted in an increase in malaria to an API of 62.2 as compared to an API of 6.5 in 2011, demonstrating the importance of high IRS coverage [6]. Recommended IRS coverage was also not achieved in Zambezi region, which is situated in the west of the country, during the 2014/2015 malaria season [9]. Thus, consequent to these factors, i.e., insufficient IRS coverage alongside IRS only functioning on insecticide-susceptible and indoor-resting vectors, it is important that the MoHSS elucidates the gaps in LLIN protection regarding where and when exposure to malaria occurs, towards the development of targeted and tailored intervention strategies.

Across recent gains and losses regarding malaria transmission in Namibia, there is a knowledge gap on the vectors responsible for ongoing malaria transmission, and human behaviors that expose humans to the vectors. The most recent data on malaria vectors in Namibia [10] were collected in 2005 in a single region (Kavango East) using limited methodologies. This suboptimal level of current baseline vector data makes it difficult to formulate optimal malaria elimination strategies. In addition, the lack of a focused, question-based surveillance framework does not allow for tailored surveillance to characterize drivers of transmission (and outbreaks) that are intrinsically dynamic in the face of a seasonal system and multiple interventions [11–16]. These drivers include vector species composition and behaviors, along with human behaviors and how the intersection of these behaviors impact intervention efficacy [17–20].

Towards answering the MoHSS’s question of “where and when are humans being exposed to bites of *Anopheles* mosquitoes?” i.e., what are the human and vector drivers of malaria transmission, as well as the gaps in protection with respect to the use of LLINs, the Entomological Surveillance Planning Tool (ESPT) [21] was applied to malaria endemic regions of northern Namibia.

## Methods

### Applying the ESPT

The ESPT [21] is a decision-support tool for planning entomological surveillance activities, interpreting entomological data, and guiding programmatic vector control decisions. The Namibia MoHSS piloted an ESPT-guided entomological surveillance plan based on priority program questions. The present study centers on one program question: “where and when are people exposed to *Anopheles* bites?” The ESPT was used to select question-based minimal essential indicators, outline a sampling design based on available capacity, and serve as a framework for data analysis and interpretation of findings [21].

### Study area

**Fig. 1:**
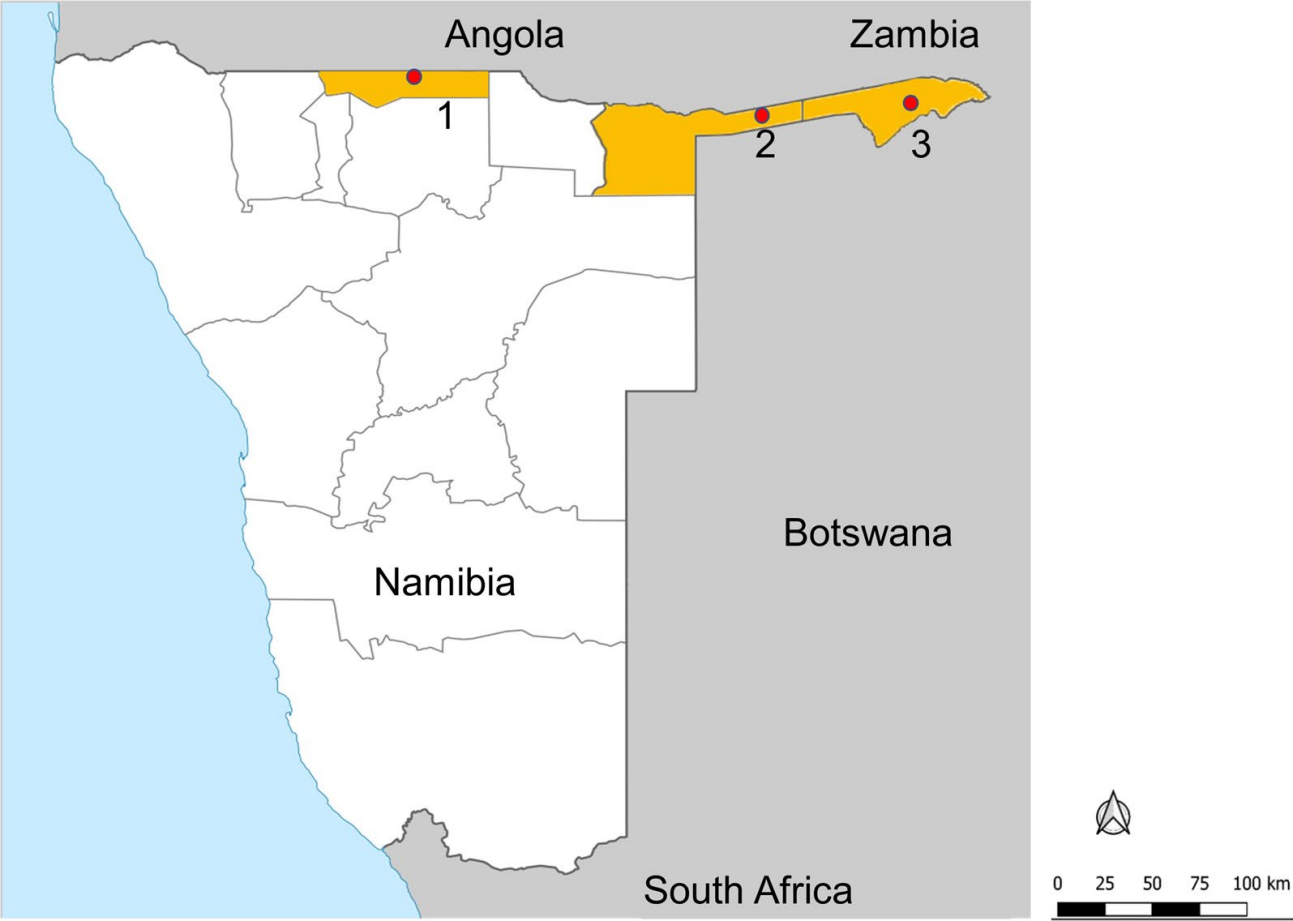
A map of the Republic of Namibia with sentinel site locations and administrative regions included in this study highlighted. *1* Okanghudi village (Ohangwena region), *2* Shadikongoro village (Kavango East region), and *3* Sibbinda village (Zambezi region)

The sentinel villages in the operational entomological surveillance were Shadikongoro village (Kavango East region), Okanghudi village (Ohangwena region), and Sibbinda village (Zambezi region) in malaria endemic northern Namibia (Fig. 1). Kavango East is a highly populated region bordering Angola to the north and Botswana to the south-east. High average annual rainfall (565 mm) and the presence of the Kavango River support the presence of marshes that serve as mosquito larval sites. Malaria transmission peaks during the rainy season (November–April) [22, 23]. Though characterized by a hot semi-arid climate, winter temperatures vary between 26 and 6 °C, with low temperatures negatively impacting mosquito populations. Ohangwena, which also borders Angola to the north, has a similar geography and malaria epidemiology to Kavango East. The temperature range is between 5 (winter) and 36 °C (summer), and the average annual rainfall is 54.8 mm. The arid, sparsely populated Zambezi region borders Zambia and Angola to the north and Botswana to the south. Seasonal malaria transmission peaks between December and April and is highly dependent on rainfall patterns; the rainy season is from November to April. The Zambezi and Kwando rivers run through this region, resulting in numerous water bodies that support vector populations. Primary interventions in these regions included the targeted distribution of LLINs and IRS with deltamethrin [6], though LLINs were not routinely distributed after 2015.

The occupation of most people in these regions is crop farming or cattle herding, with extensive movement in the latter across borders into neighboring countries. Here, the mobility-associated lack of protective items such as LLINs, along with cross-border movement into areas of higher transmission, ensure exposure to malaria infection and hence a continuous flow of parasites into Namibia [24].

### Mosquito collection

Surveillance activities were conducted during the malaria transmission season in March 2018 for eight consecutive nights. Four sentinel structures per site were selected and both human landing catches (HLCs) and human behavior observations (HBOs) were consented to for a total of 32 collection nights per site. Selected structures were representative of local constructions (with respect to building materials and size) and at least 100 m from each other. For each HLC house, a two-person team collected mosquitoes from 1800 to 0600 hours. HLC collectors were from the community but were not household members. One collector, positioned near the sleeping area of the inhabitants, sampled indoors, and the second collector sampled outdoors while sitting about 2–10 m from the house entrance. Each collection hour comprised a 50-min collection period and a 10-min break for the collectors. To minimize collection bias, the collectors switched collection position each hour and day. Each two-person team had one supervisor to verify the quality of the collections. Adult mosquitoes collected hourly were stored in individual labeled cups, then killed with chloroform and stored in individual Eppendorf tubes containing silica gel. Each tube was labeled with a code that indicated the date, household, location, and hour of collection for morphological and molecular species determination.

### Mosquito processing

The mosquitoes were transported to the laboratory and identified morphologically to species using the key developed by Gillies and Coetzee [25]; only female *Anopheles* mosquitoes were further processed. Specimens morphologically identified to the *Anopheles gambiae* complex and *Anopheles funestus* group were identified to species using polymerase chain reaction diagnostic assays [26, 27], while those that were identified as '*Anopheles* other,’ or for which there was no amplification in the polymerase chain reaction assays, were sequenced at the ITS2 and/or CO1 region towards species determination [28, 29].

### Human behavior observations

The HLC collectors documented hourly spatial presence, intervention use (i.e., LLIN), and sleeping patterns in each of the HLC houses, alongside the HLC catches. At the end of each HLC collection hour, the HLC collector positioned outside the HLC house counted and recorded (i) the number of people asleep/awake outside (peri-domestic area—within a 10-m radius of the structure), while the HLC collector positioned inside the house counted and recorded (ii) the number of people inside who were awake (not under an LLIN), (iii) the number of people asleep/resting inside under an LLIN, and (iv) the number of people inside who were asleep but not under an LLIN. Additional general behavioral categories included in the pre-populated data collection form included activities such as food preparation, eating, work, and socializing—limited to this peri-domestic area. The HLC collectors were excluded from these HBO count data [18, 28]. Data were collected on all the people present in the space and were not limited to household members only. Data were recorded on paper forms and then entered into an Excel spreadsheet; the Excel spreadsheets were then compared to the paper forms to check for accuracy.

### Data analysis

HLC-based human landing rates were used as a proxy for human biting rate (HBR), which was calculated as bites/person per hour or bites/person per night (b.p.n.). Human behavior-adjusted biting rates were calculated as in Monroe et al. [18].

## Results

To answer the NVDCP’s question to achieve a baseline understanding of where and when people were exposed to *Anopheles* bites in Namibia, HLCs were conducted alongside HBOs in malaria endemic Kavango East, Ohangwena and Zambezi regions.

### Vector species composition and bionomics

*Anopheles* species composition and bionomics were heterogenous across sites. A total of 616 mosquitoes were collected from three regions, both indoors (*n* = 253) and outdoors (*n* = 346). These included three primary vectors: *Anopheles arabiensis*, *Anopheles gambiae* sensu stricto (s.s.) and *Anopheles funestus* s.s.; *Anopheles arabiensis* was the most abundant species across all the regions. All three primary species showed a high affinity for outdoor biting. The other *Anopheles* species (pooled in *Anopheles* other) that were collected were *Anopheles coustani* sensu lato, *Anopheles squamosus*, and unidentified *Anopheles* specimens.

#### Kavango East

**Fig. 2:**
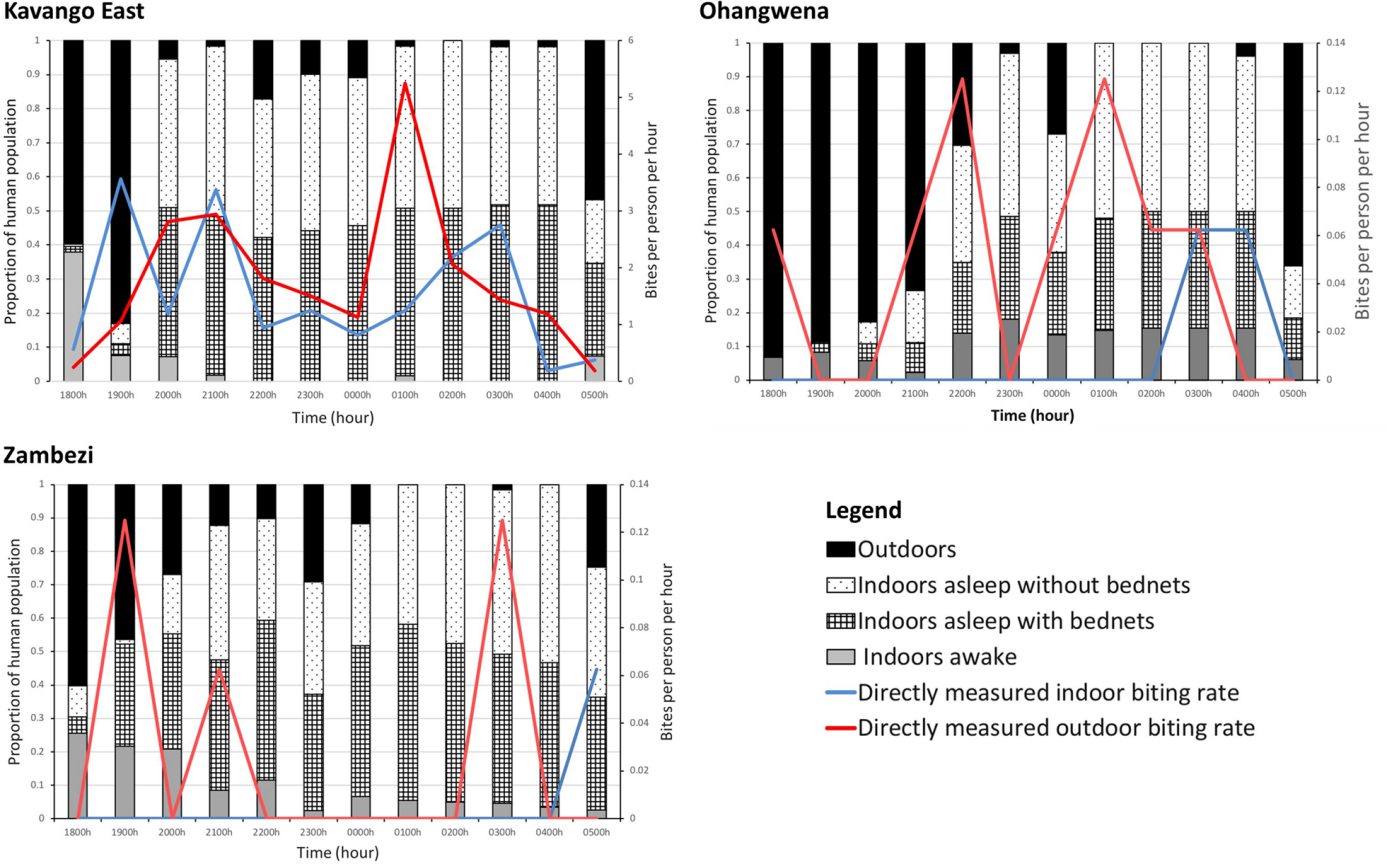
Overall *Anopheles* landing rates over the night (*lines*) and parallel human behaviors (*bars*) directly measured indoors and outdoors for Kavango East, Ohangwena and Zambezi regions

*Anopheles arabiensis* was the primary vector caught in Kavango East and demonstrated slightly higher outdoor biting rates (15.12 b.p.n. indoor, 18.68 b.p.n. outdoor; *n* = 541). This species was followed by exophagic *An. gambiae* s.s. (0.12 b.p.n. indoor, 2.5 b.p.n. outdoor; *n* = 42), then low numbers of endophagic *An. funestus* s.s. (0.5 b.p.n. indoor, 0.13 b.p.n. outdoor; *n* = 2), and other (pooled) *Anopheles* species (0.56 b.p.n. indoor, 0.31 b.p.n. outdoor; *n* = 14). Overall, *Anopheles* biting rates were 18.43 b.p.n. indoor and 21.62 b.p.n. outdoor, with host-seeking occurring throughout the night with peaks between 1900 to 2100 hours and between 0100 and 0300 hours (Table 1; Fig. 2).

**Table 1:** Species composition and biting rates by region

Region	Species	Indoor biting rate (b.p.n.)	Outdoor biting rate (b.p.n.)	Total
Kavango East	*Anopheles arabiensis*	15.12	18.68	541
	*Anopheles gambiae *s.s.	0.13	2.5	42
	*Anopheles funestus *s.s.	0.5	0.13	2
	*Anopheles* other	0.56	0.31	14
	Total	18.43	21.65	599
Ohangwena	*An. funestus *s.s.	0.13	0.5	10
	*An. arabiensis*	0.06	0.06	1
	Total	0.13	0.57	11
Zambezi	*An. arabiensis*	0	0.18	3
	*An. funestus *s.s.	0.06	0.12	3
	Total	0.06	0.31	6

*b.p.n.* Bites/person/night, *s.s.* sensu stricto

#### Ohangwena

*Anopheles arabiensis* and *An. funestus* s.s. were both found at lower densities, primarily outdoors, in Ohangwena. The *An. arabiensis* specimen was captured outdoors (0.06 b.p.n. outdoor; *n* = 1). *An. funestus* had slightly higher biting rates (0.13 b.p.n. indoor, 0.5 b.p.n. outdoor; *n* = 10). Mosquitoes were found to host seek throughout the night (Table 1; Fig. 2).

#### Zambezi

Similar proportions of *An. arabiensis* and *An. funestus* s.s. were found in Zambezi, with *An. arabiensis* found outdoors (0.18 b.p.n. outdoor; *n* = 3) and *An. funestus* s.s. found both indoors and outdoors (0.06 b.p.n. indoor, 0.12 b.p.n. outdoor; *n* = 3). Similar to Ohangwena, vectors were found to host seek throughout the night (Table 1; Fig. 2).

### Human behavioral observations

Data on human behaviors that impact exposure to mosquitoes were collected parallel to mosquito collections. Similar to mosquito behaviors, human behaviors were site specific, although they followed patterns, with people being outdoors in the evening and morning, and indoors awake or asleep (with or without LLINs). Outdoor activities were primarily related to food preparation and eating, work, and socializing.

#### Kavango East

There was an average minimum of 2.8 and a maximum of 10.6 people reported per structure (both inside and outside) per hour of data collection. People were present outdoors in the evening and early morning and moved indoors at 2000 hours to sleep. Approximately 30.68% of person-time was spent under an LLIN between 1800 and 0600 hours, with most sleep occurring between 2000 and 0500 hours. Approximately the same proportion of person-time was spent asleep not under an LLIN (29.02%). A small proportion of people remained outdoors throughout the night, socializing and/or eating (Fig. 2).

#### Ohangwena

The average number of people observed per hour per structure ranged from 1.63 to 14.9 in Ohangwena. People congregated and were present outdoors significantly more in Ohangwena than in the other regions. People moved indoors at approximately 2200–2300 hours to sleep. Only 10.0% of person-time was spent under an LLIN from 1800 to 0600 hours, with a slightly higher proportion (14.1%) spent asleep when not under an LLIN; the primary sleeping period was between 2200 and 0500 hours. In this region, people tended to congregate (working and socializing) early in the evening outdoors (Fig. 2).

#### Zambezi

The average number of people observed per hour per structure ranged from 3.75 to 8.8 in Zambezi. People were present outdoors in the evening and early morning and moved indoors at about 2000 hours to sleep. Approximately 30.6% of person-time was spent under an LLIN from 1800 to 0600 hours, with the primary sleeping period being 2000–0500 hours. Outdoor activities were primarily related to food preparation and eating, work and socializing. Small numbers of people were found outdoors throughout the night (Fig. 2).

### Human behavior-adjusted biting rates

Directly measured *Anopheles* biting rates were adjusted based on human spatial and temporal presence, factoring in LLIN use to identify periods of exposure to *Anopheles* biting [18]. Species-specific exposure varied based on vector and human behavior, with most exposure occurring outdoors for Ohangwena and Zambezi, but indoors for Kavango East (Table 2).

#### Kavango East

**Fig. 3:**
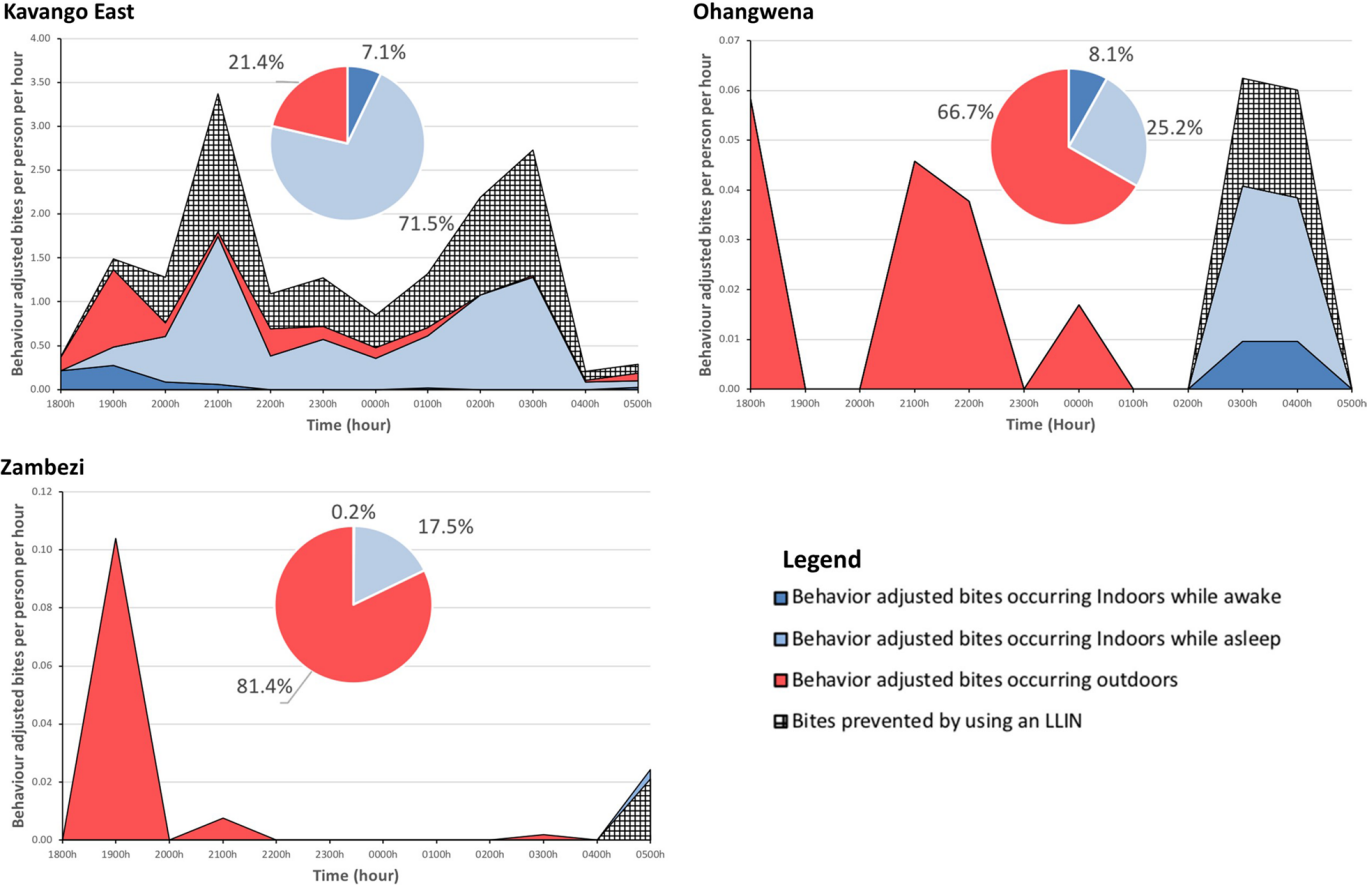
Human behavior-adjusted biting rates for all *Anopheles* along with long-lasting insecticidal net (*LLIN*)-based protection across the night for Kavango East, Ohangwena, Zambezi. Insets (pie charts) indicate proportions of exposure to *Anopheles* bites in each of the primary exposure spaces—outdoors, indoors while awake, and indoors while asleep and not protected by an LLIN. Most exposure was outdoors for Ohangwena and Zambezi, with primarily indoor exposure for Kavango East

**Fig. 4:**
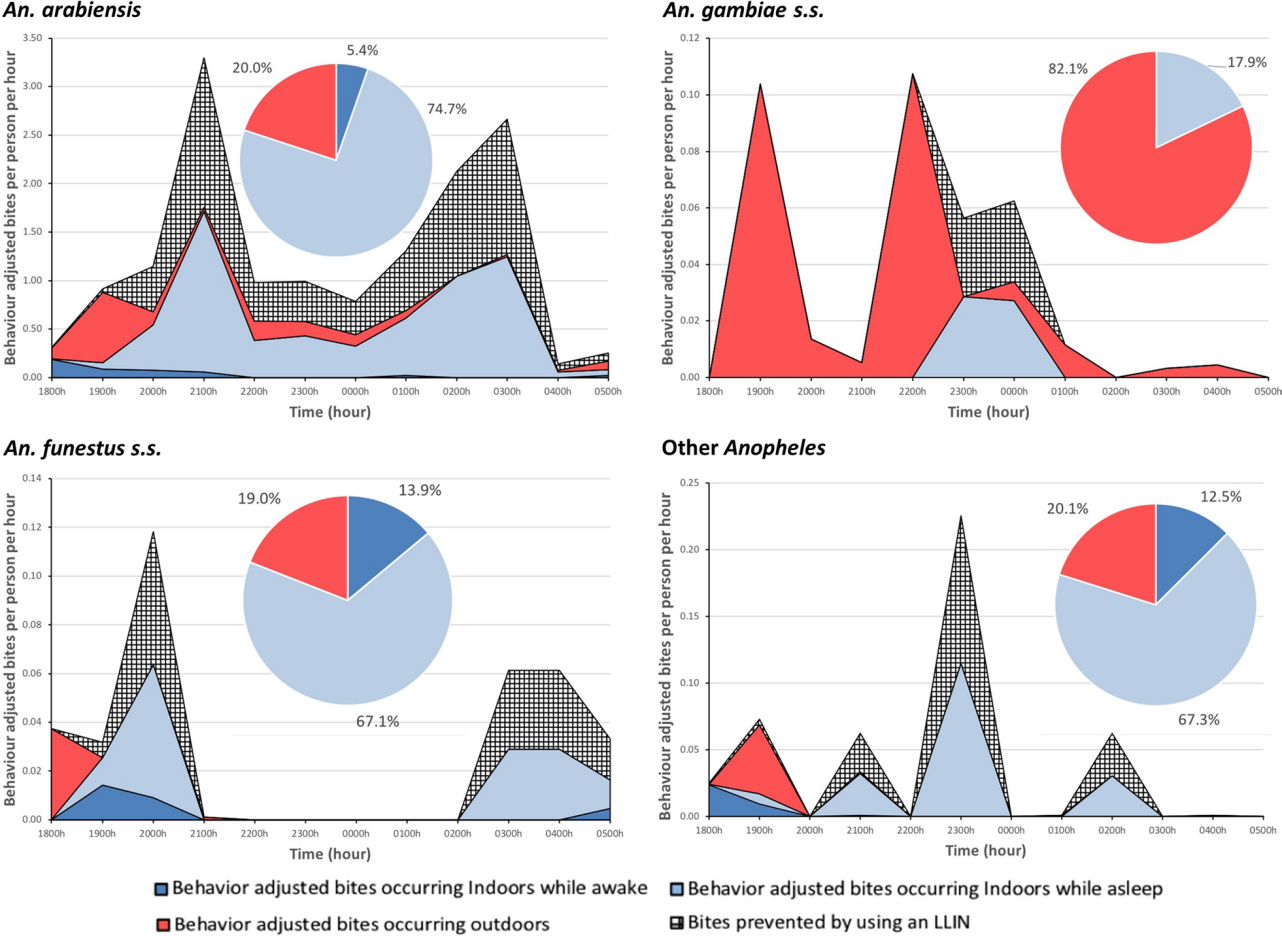
Species-specific human behavior-adjusted biting rates and LLIN-based protection in Kavango East over the course of a night. Insets (pie charts) depict human behavior-adjusted spatial exposure. Both temporal and spatial exposure to different vectors was based on vector behaviors. Though spatial exposure to *Anopheles arabiensis, Anopheles funestus* sensu stricto (s.s.) and other *Anopheles* was similar, temporal exposure varied by each species. Exposure to *Anopheles gambiae* s.s. was primarily outdoors. The proportion of bites occurring indoors for an unprotected individual awhile awake, occurring while asleep for an unprotected individual, prevented by using an LLIN, occurring indoors for a protected user of an LLIN, and occurring outdoors for each site are presented in Table 2

Overall, over the course of a night, 41.9% of bites were prevented by LLINs, based on the overlap of vector and human behaviors, including intervention use. Of the remaining exposure to bites (58.1%), 71.5% (6.83 b.p.n.) occurred indoors while asleep and not using LLINs, 21.4% (2.04 b.p.n.) occurred outdoors, and 7.1% (0.68 b.p.n.) was indoors while awake (Fig. 3). Species-specific bionomic traits impacted both exposure to bites and protection through LLINs (Fig. 4). *Anopheles arabiensis* accounted for 88.7% of exposure (8.47 of 9.55 b.p.n.), while *An. gambiae* s.s., *An. funestus* s.s. and other *Anopheles* accounted for less than 5% of exposure each.

**Table 2:** Exposure to *Anopheles* bites for each behavioral group is presented by site

Outcome measure		Kavango East (%)	Ohangwena (%)	Zambezi (%)
Protection	Proportion of all vector bites prevented by using an LLIN	72.2	18.2	15.1
Gaps in protection	Proportion of exposure occurring indoors for a user of an LLIN	5.2	5.9	0.1
	Proportion of vector bites occurring while asleep for an unprotected individual indoors	71.5	25.2	17.5
	Proportion of vector bites occurring indoors while awake for an unprotected individual	78.6	33.3	18.6
	Proportion vector bites occurring outdoors	21.4	66.7	81.4

*LLIN* Long-lasting insecticidal net

#### Ohangwena

Approximately 15.4% of exposure was prevented by LLINs, with 66.7% (0.16 b.p.n.) of remaining exposure occurring outdoors, 25.2% occurring indoors while asleep and not using LLINs, and 8.1% occurring indoors while awake (Fig. 3). Most exposure was outdoors and due to *An. funestus *s.s.

#### Zambezi

Over the course of a night, LLINs prevented 13.2% of bites. Remaining exposure was primarily outdoors (81.4%; 0.11 b.p.n.), indoors when asleep and not using a LLIN (17.5%; 0.02 b.p.n.), followed by indoors while awake (1.2%) (Fig. 3).

## Discussion

Since interventions are designed to take advantage of susceptible vector behaviors, successful control of malaria vectors largely depends on knowledge of their bionomic traits, species composition, and abundance [30]. Human behaviors also factor in as drivers of intervention efficacy; this is often evaluated on the basis of LLIN usage, but should also be determined by the spatial and temporal presence of humans alongside that of vectors [18–20, 28]. Towards a baseline understanding of where and when people were exposed to *Anopheles* bites in Namibia, the MoHSS along with the University of Namibia (UNAM), the Clinton Health Access Initiative (CHAI), and the Malaria Elimination Initiative, implemented the ESPT [21], with a focus on determining gaps in protection towards developing optimal strategies that may counter residual transmission in Namibia.

The ESPT-guided operational entomological surveillance with molecular validation confirmed the presence of several primary vectors—*An. gambiae* s.s., *An. funestus* s.s., and *An. arabiensis*. *Anopheles arabiensis* was the most abundant species overall (Table 1). A subset of secondary vectors (grouped as ‘*Anopheles* other’) demonstrated the presence of *An. coustani*, *An. squamosus*, and a member of the *An. coustani* group (*Anopheles* cf. *coustani 1* NFL 2015 [28]) (Table 1). The presence of the primary vector *An. gambiae* s.s. was a significant finding, as the MoHSS has not documented its presence [10,31] since La Grange putatively described it in 1988 [32]. The identification of this primary vector species along with secondary vectors points to the importance of developing and sustaining in-country molecular capacity, which is supported in Namibia by UNAM. The differences in species composition and abundance across the three regions also highlight the importance of conducting routine entomological surveillance across the sentinel sites to monitor these changes across time and location. This allows for better tailoring of interventions for each region.

This snapshot of vector bionomics shows species- and geography-specific indoor and outdoor exposure (Fig. 2). Interestingly, the few *An. funestus* s.s. sampled in Ohangwena were exophagic—contrary to the literature [11, 33–35]. By and large, vectors were host seeking both indoors and outdoors, which indicates the potential for both indoor and outdoor exposure. Additional sampling sites may allow a deepening of knowledge on species-specific behaviors. Site-specific human behaviors varied and consequently dictated exposure.

In 2012, the Namibian government set a goal to achieve 95% LLIN coverage for the entire population and not merely vulnerable groups. Over 625,000 LLINs were distributed at health facilities, outreach sites, antenatal clinics and through mass campaigns [2]. In 2014, a total of 87,900 LLINs were distributed in Zambezi, Kavango and Omusati, as these regions had the highest malaria caseloads in the country [2]. In 2015, approximately 800,000 LLINs were distributed to vulnerable groups in Kavango East, Kavango West, Ohangwena, and Kunene regions [6], although it is not clear whether the coverage recommended by the World Health Organization (WHO) was attained. It was reported that these LLINs did not offer any added protection [6], and distribution efforts were thus discontinued. The consequences of LLINs not being the primary intervention in Namibia were low levels of access to the nets and therefore use, i.e., 20% in Kavango East, 31% in Zambezi, and 14% in Ohangwena, which are considerably below the 80% coverage recommended by the WHO for community protection [36]. This also explains why most of the indoor exposure in all three regions in the present study was a consequence of people not using LLINs when asleep (Fig. 3). Protection with LLINs may be significantly increased with targeted LLIN distribution alongside an education campaign. Use of LLINs could lead to a reduction in indoor exposure of up to 71.5% in Kavango East, 25.2% in Ohangwena and 17.5% in Zambezi. The other primary exposure space was outdoors, due to the presence of both humans and outdoor biting vectors. The contribution of human behavior to this type of exposure was particularly demonstrated in Ohangwena, where high proportions of people outdoors throughout the night increased exposure, and may be a driving factor of malaria in this region, despite low vector biting rates. For example, there were up to 60 people present outdoors per night for the four houses examined, with a total of 238 people over the four nights of data collection, all within the first hour (1800–1900 hours). In addition, village-based evening and night-time social behaviors—including cooking and eating, and social activities such as drinking alcohol—facilitate exposure to mosquito bites. Given this, additional interventions outdoors may be necessary to reduce exposure outdoors and drive down transmission. Note that raw vector landing rates were different from human-adjusted exposure (Fig. 2 vs. Fig. 3), demonstrating the importance of factoring in the human component of exposure [17–19].

Species-specific vector bionomic traits can also guide the tailoring and targeting of interventions. This was demonstrated in Kavango East, where the three primary vectors (*An. arabiensis*, *An. gambiae* s.s. and *An. funestus* s.s.) along with secondary vectors (other *Anopheles*) demonstrated different behaviors and hence exposure profiles. Even though *An. arabiensis*, *An. gambiae* s.s. and other *Anopheles* had similar exposure profiles, with greater exposure indoors (Fig. 4), temporal exposure and the amount of exposure varied based on human behavior profiles and HLC landing rates (Fig. 4). The exposure profile for *An. gambiae* s.s. differed markedly from those for these species, again due to species behavior, as its exposure was primarily outdoors. These observations demonstrate that vector bionomic traits have a significant impact on exposure when human behavior and intervention use are factored in. Since geographic variation in species-specific traits (Table 1) might also impact exposure profiles, local mosquito bionomic traits need to be factored in when considering how appropriate an intervention is.

The current primary intervention in Namibia (IRS) targets indoor resting mosquitoes, allowing indoor and outdoor biting when people are not protected [11, 30, 33, 37]. Consequent to insufficient IRS coverage [6, 9] alongside IRS only functioning on insecticide-susceptible and indoor resting vectors, it was considered important that the MoHSS gain an understanding of the gaps in protection, i.e., where and when exposure to malaria continued to occur. The term gap in protection is used to describe a circumstance when an individual and/or household is potentially exposed to malaria infection (i.e., an infective mosquito bite) due to a lack of effective and/or adequate preventive intervention in place to reduce exposure to mosquito bites. Gaps in protection can be directly identified through the assessment of how local human and vector behaviors interact with interventions [21].

In the three regions, the ESPT was utilized to describe and quantify gaps in protection. A primary and significant observation is the site-based gap in protection based on lower than optimal LLIN usage—an outcome indicative of the targeted LLIN strategy in contrast with universal coverage. Increasing the distribution of LLINs alongside optimizing LLIN use, and therefore efficacy, has the potential to significantly impact malaria transmission. Other identified gaps in protection include indoor exposure before sleep (outside the protection of LLINs) and outdoor transmission where suitable interventions may include larviciding, spatial repellents [38–40], topical repellents, or other supporting vector control interventions. LLINs used alone may be insufficient for the elimination of malaria based on where and when exposure to mosquito bites have been documented. Understanding the extent to which IRS (the primary vector intervention in Namibia) impacts exposure to *Anopheles* bites, along with drivers of IRS success (coverage, acceptability, insecticide resistance, duration of effect) would help researchers elucidate the extent to which these interventions function together, as well as remaining gaps in protection.

Operational surveillance directed towards programmatic questions has innate limitations (relative to academic research) due to capacity and financial limitations, as is the case across many malaria endemic countries. The use of only four structures for both HBOs and the determination of indoor and outdoor landing rates (using HLCs), and only one site per region, meant that the sample size was less than optimal. Caution has to be used when generalizing these findings to the regional level or when making comparisons across regions. Increased funding and capacity would have enabled a greater spatial and temporal sampling frame with statistical analysis. Understanding access to LLINs would also have enabled a greater understanding of coverage gaps. Another limitation with respect to answering the main question—where and when are people exposed to *Anopheles* bites?—is only having data from domestic and peri-domestic settings. Capturing the time spent by people and their related behaviors away from household structures is an important part of gaining an overall picture of exposure, especially given occupation and population mobility considerations [18–20].

## Conclusions

The MoHSS-directed, question-based surveillance framework was implemented in an operational context with the aim of generating a focused snapshot of human and entomological drivers of malaria transmission from integrated data on vector and human populations and their behaviors to inform programmatic decision making [21]. The focused, ESPT-based surveillance strategy used here allowed us to use limited resources optimally and to answer the MoHSS question to assist programmatic decision making. The evidence presented here of spatial and temporal heterogeneity in bite exposure due to human- and vector species-specific behaviors demonstrates where gaps in protection exist and how current LLIN use can be optimized.

## Data Availability

The data that support the findings of this study are available from the Ministry of Health and Social Services, Namibia but restrictions apply to the availability of these data, which were used under license for the current study, and so are not publicly available. Data are however available from the authors upon reasonable request and with permission of the MoHSS.

## Abbreviations

API: Annual parasite index
b.p.n.: Bites per person per night
CHAI: Clinton Health Access Initiative
ESPT: Entomological Surveillance Planning Tool
HBO: Human behavior observation
HLC: Human landing catch
IRS: Indoor residual spraying
LLIN: Long-lasting insecticidal net
MoHSS: Ministry of Health and Social Services
NVDCP: National Vector-borne Disease Control Program
s.s.: Sensu stricto
UNAM: University of Namibia
